# Stakeholders’ Roles and Views in the Provision of Sexually Transmitted Infection Services Among Key and Priority Populations in Limpopo Province, South Africa

**DOI:** 10.3390/healthcare13182262

**Published:** 2025-09-09

**Authors:** Mohlago Ablonia Seloka, Edith Phalane, Refilwe Nancy Phaswana-Mafuya

**Affiliations:** South African Medical Research Council/University of Johannesburg Pan African Centre for Epidemics Research Extramural Unit (SMRC/UJ PACER), Faculty of Health Sciences, University of Johannesburg, Johannesburg 2006, South Africa; edithp@uj.ac.za (E.P.); refilwep@uj.ac.za (R.N.P.-M.)

**Keywords:** Limpopo province, primary healthcare facilities, sexually transmitted infections, South Africa, key and priority populations

## Abstract

**Background:** There is a dearth of evidence on the roles and views of stakeholders regarding the sexually transmitted infections (STIs) service provision among key and priority populations (KPPs) within primary healthcare (PHC) settings. **Aim**: This study assessed the roles and views of stakeholders regarding the STI services scope, content, accessibility, quality, affordability, and availability, as well as associated gaps and successes among KPP within PHC facilities in the Capricorn District of Limpopo Province in South Africa. **Methods**: An exploratory research design was used. In-depth face-to-face interviews with 18 STI stakeholders were conducted. The STI stakeholders were purposively selected from five PHC facilities. An inductive analytical approach was employed to develop themes and sub-themes. Tesch’s step analysis informed the development of the thematic analysis process. **Results**: The presence of peer counsellors, home-based caregivers, and the operation of STI services day and night in two selected facilities enhanced access and availability of STI services. Consistent in-service training for service providers was implemented to improve service quality and maintain professional competency. Barriers that prevented adequate STI service provision in this study included staff shortages, inadequate filing systems, lack of advanced-diagnostic equipment, and patients’ noncompliance with treatment regimens. The successes of the STI service provision were effective STI treatment and services integration within the facilities. **Conclusions**: The findings of this study have unveiled several methods to increase access and availability to STI services among KPPs in the selected PHC facilities. We recommend gathering responses and experiences from STI service users regarding the current STI service provision to foster innovative and targeted approaches within PHC facilities in Limpopo Province.

## 1. Introduction

Sexually transmitted infections (STIs) are not only preventable, but also treatable. However, when they remain undiagnosed or untreated, they can lead to significant health complications [1]. Key and priority populations (KPP) are populations who, because of risky sexual conduct and multiple sexual partners, have a higher likelihood of acquiring and transmitting STIs [2]. These populations include “female sex workers (FSWs) and their clients, males who have sex with men (MSM) and their partners, people who use drugs (PWUD), pregnant women, incarcerated individuals, transgender people (TG), adolescent girls, young women (AGYW), and offenders in prison settings, people with mental disorders and physical disabilities” [2,3]. In South Africa, the most prevalent STIs presented in primary healthcare (PHC) facilities among men are male urethritis syndrome (MUS) [4]. According to Kularatne [4], the MUS incidence among men was mostly caused by *Neisseria gonorrhoeae* (NG) (87.8%) followed by *Chlamydia trachomatis* (CT) (21.0%). Approximately 24.7% asymptomatic MSM had extragenital STIs, and 9.4% had high nontreponemal antibody titres; however, the pharyngeal STIs were less prevalent among this population [5]. Jones et al. [6] reported a higher rectal incidence of 29.7/100PY among MSM and 30.3/100PY among TG women. As for CT STI, young women aged 18–21 years have indicated a higher prevalence compared to women aged 26–33 years [7]. On the other hand, *Trichomonas vaginalis* (TV) STI incidence was higher in the 26–33-year age group compared to the 18–21 and 22–25-year age groups [7]. Among pregnant women attending antenatal care (ANC) in South African PHC facilities, syphilis was observed to be the predominant STI, with 3.1% (95% CI: 2.9–3.3) reported cases in 2022 [8]. It has been reported that untreated syphilis in pregnancy can potentially lead to serious adverse health outcomes such as neonatal death, low birth weight, stillbirth, congenital syphilis, neurological issues and miscarriage [9]. Hence, proper and timely management of STIs, including syphilis, in PHC facilities is imperative. Studies conducted on STI among FWS are scarce, especially in South African PHC facilities. Meanwhile, FSW in Zimbabwe had a high proportion of infections that were both asymptomatic and clinically undetectable (gonorrhoea: 41.2%, chlamydia: 51.7%, trichomonas: 62.8%) [10].

Approximately 84% of the South African population, including KPP, seek healthcare services mainly from the PHC facilities where syndromic management is the principal treatment method [11]. The syndromic management approach was established to overcome the lack of financial support, the need for laboratory-based resources and individuals with expertise in resource-limited settings [12,13]. The syndromic management approach increases access to healthcare services in PHC facilities, and patients receive access to medical history, screening, diagnosis, treatment, condom provision, counselling and partner referral all at once [11]. In a qualitative study conducted in Kenya, exploring healthcare provider perspectives on managing STIs, it was reported that there were frequent stock-outs of STI medications [14]. The study also found that healthcare workers used the syndromic approach for managing STIs, where additional services, such as counselling and enough condoms, were provided during the clinic visit [14]. Another study conducted in the eThekwini district municipality among nurses found that improved access, awareness, and knowledge of STIs among study participants was through the use of eHealth methods (SMS, email), health education, and community-led strategies [15]. The study further outlined challenges, such as the clash between the clinics’ operating times and the university and school’s operating times [15]. The author stated that by the time students or learners are back, the clinics would have been closed, and this would limit access to STI services [15].

Moreover, KPPs were reported to encounter significant barriers when seeking screening and treatment for STIs, such as violence, stigma, discrimination, human rights violations, and a bad attitude from healthcare workers (HCWs), which is in stark contrast to the general population [2,3,13]. According to the South African National Strategic Plan (NSP) 2023–2028 [2], structural barriers hinder KPP’s access to necessary healthcare services. Research by Makura et al. [16] also highlighted that these barriers negatively impact healthcare-seeking behaviour among KPPs, potentially worsening health outcomes and contributing to a higher prevalence of STI. Therefore, there is a need to address all the challenges and barriers that prevent equitable access to STI healthcare services in PHC facilities to reduce daily STIs transmissions [2,17].

Nonetheless, there are limited studies carried out on stakeholders’ roles and views on STI services provision in PHC facilities in Limpopo Province. Therefore, this study sought to understand stakeholders’ roles and views in STI services availability, accessibility, affordability, scope, content, and quality delivered among KPPs in PHC facilities in Limpopo Province of South Africa. Additionally, it explored the gaps and successes in STI services provision.

## 2. Materials and Methods

### 2.1. Study Design

An exploratory, descriptive research design was used to evaluate the roles and views of STI stakeholders regarding availability, accessibility, affordability, and quality, as well as the associated gaps and successes.

### 2.2. Study Setting

The study was based in Polokwane City in the Capricorn district, Limpopo Province of South Africa. It included four clinics and one community healthcare centre in Polokwane. More detailed information on the study setting has been previously published. The study obtained ethical approval from the University of Johannesburg, Faculty of Health Science, Research Ethics Committee [REC-2589-2024] [18].

### 2.3. Sampling and Data Collection

#### 2.3.1. Sampling

Purposive sampling was utilised to choose the five PHC facilities. A total of 18 stakeholders were recruited to take part in this study across the five PHC facilities. Three stakeholders, namely, professional nurses or clinicians and facility managers, were purposively selected within each PHC facility. The sampling method was chosen to leverage the stakeholders with relevant STI expertise and experience to provide context-rich information on STI service provision among KPPs [18,19]. In this study, the terms “STI stakeholders” and “service providers” are used interchangeably. The STI stakeholders are a combination of STI service providers and policymakers, while service providers are healthcare professionals who deliver medical services to patients in PHC facilities. Policymakers are individuals involved in STI service provision outside of PHC facilities. This includes roles, such as working in STI laboratories, participating in the country’s STI surveillance efforts, and overseeing and directing the national STI and key populations services. In this study, a diploma is defined as a qualification that typically takes 2 to 3 years to complete. It focuses on practical skills and provides more specialised training. In contrast, a degree is a qualification that usually takes 3 to 4 years to complete. It offers a broader and more in-depth academic programme [20]. Additionally, screening was defined as a procedure that evaluates patients who may or may not show symptoms to assess their risk of contracting a specific infection, while testing, on the other hand, involves collecting biological samples, such as blood, saliva, urine, and swabs [21]. These samples are then analysed using laboratory equipment to confirm the presence of a specific disease [21].

#### 2.3.2. Data Collection and Recruitment

The study derived questions from a validated WHO assessment checklist tool and was also guided by NSP 2023–2028 for HIV, TB, and STIs [2,22]. The questionnaire was divided into three sections; section A was the demographic information of the STI stakeholders. Section B was the STI services provision within PHC facilities, in terms of availability, accessibility, affordability, and quality, as well as the associated gaps and successes. Section C comprised open-ended questions from the WHO pre-assessment checklist tool. The questions focused on: (A) “Is there a need to assess the surveillance system?” (B) “Is there political support to assess the surveillance system?” (C) “Is there technical capacity to conduct the STI surveillance assessment and analyse the results?” (D) “Is it likely that STI surveillance assessment outcomes will be acted upon?” (E) “Are there potential partners and collaborators who would be useful in conducting STI surveillance assessments?” [22].

The interviews were recorded using a tape recorder, and additional notes were taken and written down during the interviews. Probing questions were also used to gather in-depth information about the STI service provision during the interviews. The interviews were conducted from 20 October 2024 to 20 December 2024.

##### Recruitment

Before the interviews, the researcher (MS) contacted each facility’s operational manager, who then assisted in extending the invitation to interested stakeholders. The interested stakeholders scheduled an appointment with the first author (MS). The first author (MS), then met and shared detailed information about the study’s objectives and procedures with the study participants. The first author (MS) conducted face-to-face, one-on-one, in-depth interviews with the interested STI service providers and policymakers using a guiding questionnaire. The interviews took place in a private room in each healthcare facility. Each interview was scheduled at a time convenient for the interviewees to avoid disrupting their daily work-related routines. The stakeholders voluntarily submitted a signed written informed consent form and gave permission to be audio-recorded before taking part in the interviews. Participation in the study was entirely voluntary, and individuals could withdraw at any time before the interviews. Beyond the interview process, the study remained anonymous.

Data collection continued until we reached a point of saturation, meaning that no new insights were discovered [23]. For example, interview transcripts were reviewed and coded after data collection from each facility to identify recurring themes and sub-themes. Through a thorough ongoing analysis of themes from other facilities, it was observed that no new patterns, themes, or insights were emerging from the newly collected data provided by the service providers. This indicated that we had reached our data saturation point [23].

#### 2.3.3. Inclusion and Exclusion Criteria

Inclusion criteria involved STI service providers and stakeholders aged 18 years and above, with a minimum of one year of experience working in a PHC facility or managing STI services. The study involved stakeholders who worked in PHC facilities in Polokwane, Limpopo Province, who were willing to consent in writing and agreed to be audio recorded. Exclusion criteria included minors and individuals without experience in STI services provision [18]. Moreover, the study further excluded service providers who could not make time for the interviews due to busy work-related daily routines.

### 2.4. Data Analysis

The English language was used to conduct the interviews. The first author (MS) initiated the coding process by developing themes, using an inductive analytical approach. The external co-coder followed an inductive analytical approach and applied the eight steps of Tesch’s [24] to guide the process of developing themes and sub-themes. The steps were as follows: (i) transcripts were reviewed three times to obtain a broad understanding of the data and reflection of the participants; (ii) the stakeholder responses that stood out from the data were highlighted; (iii) recurring themes and patterns that emerge from the participant’s views were listed and grouped, (iv); emerging topics were categorised into primary, secondary, and overlapping themes and codes were written next to relevant segments (v); the data was arranged systematically for further analysis, (vi); the data was grouped according to the relevant category, (vii); the themes and subthemes were named and defined, then forwarded to the external co-coder for verification, validation and feedback; and (viii) after the agreement with the external co-coder, the data was refined, recorded, and prepared for analysis. In the final analysis, compelling, vivid themes and sub-themes were selected and compared back to the aim of the study, ensuring consistency. The manuscript was developed afterwards. No software was used for analysing themes and subthemes.

### 2.5. Data Management

The data collected from the participants was managed with strict security measures. Field notes, recordings, and transcripts from the interviews were kept strictly confidential. De-identified electronic data were stored with password encryption to avoid re-identification or linking the data to participants. According to the University of Johannesburg’s policy on data management, the collected data will be kept for five years before being securely destroyed following standard procedures.

### 2.6. Validity, Reliability, and Trustworthiness

The reliability of the data was ensured by using the same questionnaire for all study participants across the five facilities [25]. Furthermore, the validity of the information collected was strengthened through consultations with various stakeholders involved in STI services in the selected PHC facilities. Each interview transcript was recorded verbatim, following the same procedure and conversational mode to maintain methodological rigour and precision. To further enhance validity, a validated interview guide and tool from the WHO was employed. In addition, inter-coder reliability was established through collaboration between the internal coder (MS) and an external co-coder, who adhered to Tesch’s eight steps of data analysis when coding the interview transcripts. The coder and external co-coder then compared and discussed their coding outcomes until they reached consensus.

The trustworthiness of the collected data was upheld through the four key epistemological principles: confirmability, credibility, transferability, and dependability [26,27]. Confirmability was applied to improve the accuracy, plausibility, and reliability of coding through Tesch’s eight steps, as well as thorough verification and validation of the selected themes with the co-authors and an external coder.

Dependability was safeguarded by providing all data collection materials, including how the questionnaire was constructed and data collection and analysis of themes and subthemes. To establish credibility, only experienced STI stakeholders were interviewed to gather more comprehensive insights into the experiences and perspectives on the STI services provision in PHC facilities. Transferability refers to the degree to which the study outcomes can be applied in different settings. Detailed methodological procedures, especially on how themes and subthemes were developed, were provided to ensure this principle, thereby making it easier for it to be applied in other studies.

## 3. Results

### 3.1. Characteristics of the STI Stakeholders

Table 1 highlights the characteristics of the STI stakeholders. Thirty-nine per cent of the stakeholders were over 50 years old (*n* = 7), compared to those over 60 (*n* = 2), with a minimum age of 30 and the highest age of 64 years old. Most stakeholders were predominantly female (94%), compared to males (5.5%). With regard to stakeholder level of education, most of them had a diploma (49%), followed by those with a degree (37%) and those with only grade 12 education (6%). The study mainly consisted of professional nurses (50%), compared to those whose job titles were lab manager (6%), epidemiologist (6%), and deputy director (6%). Approximately 44% of the participants had 1–10 years of experience, compared to those with over 30 years of experience (6%).

### 3.2. Pre-Assessment Preparation Using the WHO STI Assessment Checklist Tool

**Figure 1:**
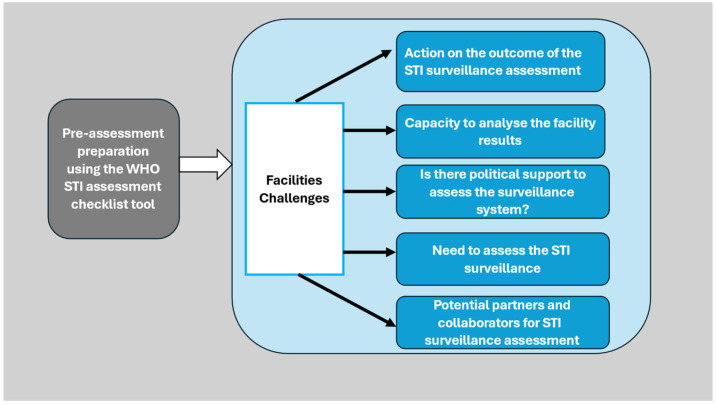
Theme and subthemes derived from the pre-assessment preparation using the WHO STI assessment checklist tool (STI—sexually transmitted infections, WHO—World Health Organisation).

The pre-assessment of the STI surveillance generated five subthemes, which were grouped as a facility challenges theme, as shown below (Figure 1).

Theme 1: Facility challenges

Service providers added that the facilities needed the capacity to carry out the assessment, analyse the results, obtain political support and receive the outcome of the STI programme assessment.

Sub-theme 1: *Action on the outcome of the sexually transmitted infections surveillance assessment.*

Service providers reported that, although the government can come to their facilities to evaluate the programmes, no outcome results would be implemented.

*“Yes, the government came to our clinic and did the assessment, but it would end there. We will never hear from them again.”*

*(P5, Female, Facility Manager, 51 years).*

Sub-theme 2: Capacity to analyse the results

Service providers reported that data analysis is not conducted at the facilities; instead, it is carried out at the district level by district officials.

*“We report our daily cases on our clinic register. Do you know what we do? At a PHC facility level, we just compile monthly summary statistics and send them to the district. For example, we compiled statistics to show how many cases there were this month. However, the report will be analysed at the district level and not by the facility.”*

*(P8, Female, Professional nurse, 48 years old).*

Sub-theme 3: Is there political support to assess the surveillance system?

Service providers explained that they have no political support to assess the STI programme within PHC facilities.

*‘No, I think all that we want now is a political buy-in and also a multi-sectoral approach and support for STI programme assessment in PHC facilities.’*

*(P18, Female Deputy Director for STIs and key population, 51 years old).*

Sub-theme 4: Need to assess the STI surveillance

Service providers believe that the STI surveillance assessment must be conducted to improve service provision among KPPs.

*’Yes, there is a big need because STIs are not prioritised as HIV; when HIV was still a pandemic, they used to talk about it together with other STIs. Now that HIV is manageable with treatment, they are no longer talking about it. They only talk about STI when they see their prevalence increasing.’*

*(P5, Female, Facility Manager, 51 years old).*

Sub-theme 5: Potential partners and collaborators for the STI programme assessment

STI stakeholders reported that the Department of Health is an implementing partner to several institutions that assist in maximising STI service provision within the PHC facilities.

*‘We have implementing partners such as the WHO, the University of Witwatersrand Reproductive Health and HIV Institute (WITS RHI), the National Institute for Communicable Diseases (NICD), the Centre for the AIDS Programme of Research in South Africa (CAPRISA) and the South African universities with which we work closely to improve the STI surveillance. These institutions assist in facilitating early diagnosis, which prompts treatment and disease surveillance in the country.’*

*(P 17, Female, Laboratory Manager, 51 years old).*

### 3.3. Stakeholders’ Views on STI Service Provision Among Key and Priority Populations

**Figure 2:**
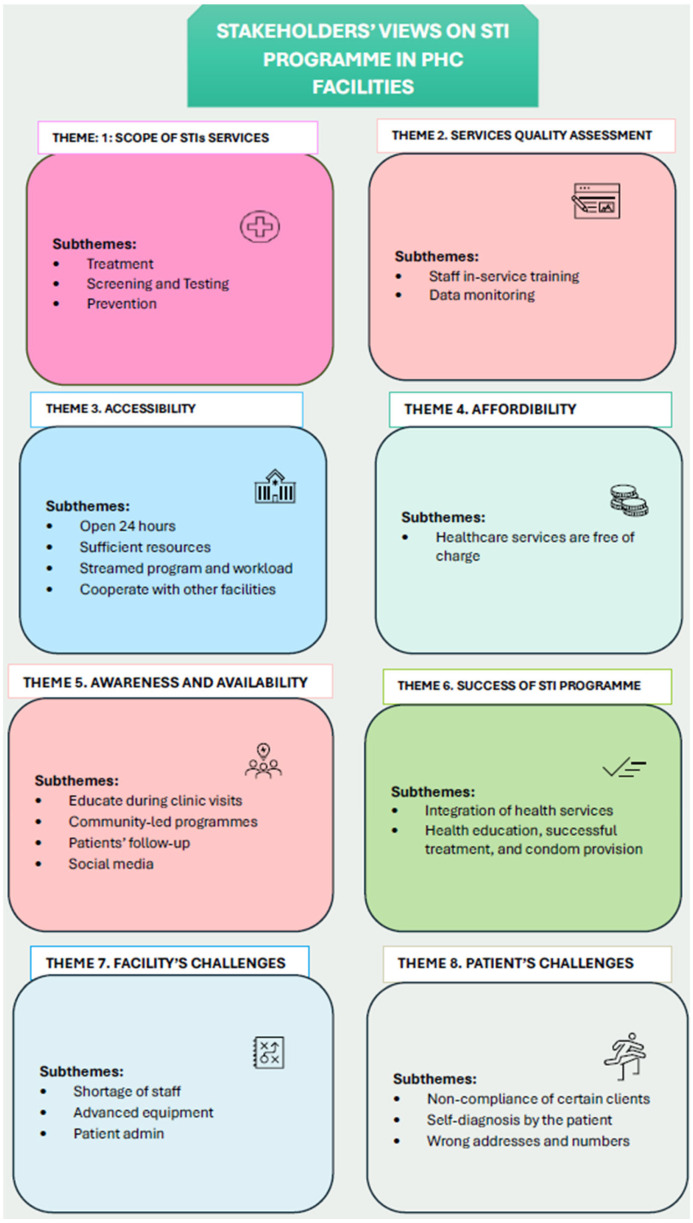
Stakeholders’ views on sexually transmitted infections services provision. (STIs—sexually transmitted infections).

Eight major themes and twenty-six sub-themes (see Figure 2) emerged from stakeholders’ perspectives on STI services.

Theme 1: Scope of STI services

The scope of the STI services within the PHC facilities included testing, screening, treatment, and prevention. Service providers indicated that they used syndromic management guidelines to assess gonorrhoea, MUS, and herpes simplex virus. They used a rapid test to test and diagnose syphilis and HIV. Antibiotics were given as injections or tablets for STI treatment. Methods such as health education, condom provision, VMMC, pap smear, counselling, and partner referrals or notifications were used to prevent STIs.

Sub-theme 1.1: Treatment

Service providers indicated that they used syndromic management guidelines or algorithms to treat STIs.

*“We treat patients with three medicines: azithromycin, ceftriaxone, and metronidazole. For genital discharge (GD) such as vaginal discharge syndrome (VDS), NG (drop), MUS, metronidazole tablets 2 g single dose, 250 mg ceftriaxone injectable, and 1 g of azithromycin single dose are used. For syphilis, we give them benzylpenicillin injectable 2.4 mu once weekly for three weeks.”*

*(P12, Female, Facility Manager, 53 years old).*

Sub-theme 1.2: Screening and Testing

Service providers indicated that they test for STIs such as HIV/syphilis, cervical cancer, and MUS.

*“We use a rapid test kit to test for HIV/syphilis, and we screen for cervical cancer as well as MUS.”*

*(P13, Female, Clinical Nurse Practitioner, 64).*

Sub-theme 1.3: Prevention

Healthcare workers explained that counselling and health education, condoms, and pap smears were used as prevention methods within PHC facilities.

*“We do not do counselling separately from our PHC services; the person is counselled before being tested. We also provide health education; we encourage our patients to use condoms consistently and undergo regular pap smear.”*

*(P2, Female, Professional Nurse, 35 years old).*

Theme 2: Services quality assessment

Service providers regularly engage in in-service training amongst themselves. This training is prompted by new guidelines, the discovery of new information by any service providers, or attendance at workshops by some members.

Sub-theme 2.1: Staff training in-service training

Service providers showed that they rely on in-service training within their facilities to enhance service provision.

*“We do regular in-service training in the facility. This can occur on a daily basis if one of the sister nurses finds new information, attends a workshop, or there is a new guideline, and we share it with others or amongst ourselves.”*

*(P7, Female, Professional Nurse, 34 years old; P12, Female, Facility Manager, 50 years old).*

Sub-theme 2.2: Data monitoring

Service providers described how they reported their data and how it was monitored in PHC facilities.

*“We report STI cases monthly; we use the registers and the system (e-tick register) to check the monthly STI average. For the completeness of the data, we use patients’ files, clinic registers, and electronic systems, checking, validating, and comparing if everything from the patients’ files and registers tallies with what is on the e-tick register.”*

*(P9, Female, Facility Manager, 50 years old).*

Theme 3: Accessibility

The PHC facilities and STI policymakers employ various strategies to ensure that STI services are accessible to all marginalised populations, and these methods are detailed in the following sub-themes.

Sub-theme 3.1: Open for 24 h

STI service providers from two selected PHC facilities indicated that they operate 24 h a day, from Monday to Sunday, to ensure that those seeking medical attention are assisted at any time.

*“All our services are available for 24 h, and our clinic operates from Monday to Sunday.”*

*(P3, Male, Facility Manager, 58 years old; P5, Female, Facility Manager, 53 years old).*

Sub-theme 3.2: Sufficient resources

STI service providers confirmed having resources for blood samples, rapid tests and enough STI medicines. Additionally, stakeholders working in the laboratory highlighted the equipment they currently use and have at their disposal.

*“Treatment for STIs is always available. For example, for ANC, we have a new test kit called a rapid test, an HIV/STI test kit. We can also withdraw blood and send it to the laboratory, and get the lab results after 72 h. The National Health Laboratory Services (NHLS) Courier guy collects blood samples or specimens daily.”*

*(P13, Female, Clinical Nurse Practitioner, 64 years old).*

*“We have a microbiology laboratory with molecular techniques to detect all the different types of pathogens causing any STI syndromes and perform the serological tests. We have our in-house assay, a multiplex PCR, Ribonucleic Acid (RNA) based, Deoxyribonucleic acid (DNA) based methods and some commercial kits that we use to verify or confirm results.”*

*(P16, Female, Epidemiologist, 47 years old; P17, Female, Lab Manager, 51 years old).*

Sub-theme 3.3: Streamed programme and workload

Stakeholders shared that they use various strategies to categorise patients, based on healthcare needs and specific services they seek from the clinics. This approach helps them organise their daily operations.

*“We use a triage strategy; we start with patients who are very sick, then minor ailments (minor illnesses and injuries), followed by babies and children.”*

*(P8, Professional Nurse, Female, 48 years old).*

Sub-theme 3.4: Cooperation with other facilities

STI service providers explained that they rely on neighbouring facilities for support during a medication stockout.

*“We give three drugs for treatment. Sometimes, you may discover that one of the drugs is unavailable, so we outsource from nearby clinics. There is a point at which, as clinics, we exchange and request medication from other clinics.”*

*(P7, Female, Professional Nurse, 34 years old).*

Theme 4: Affordability

The STI stakeholders have indicated, in this theme, that the Provincial Government is responsible for all the costs in the PHC facilities, making all the services available free of charge.

Sub-theme 4.1: Healthcare services are free of charge.

STI stakeholders have confirmed that all the services provided in the PHC facilities, including those related to STI treatment, are offered free of charge.

*“All the services in public facilities are free of charge.”*

*(P17, Female Lab Manager, 51 years old; P19, Female STI and key populations Director, 55 years old).*

Theme 5: Awareness and availability

Stakeholders affirmed that they play a vital role in increasing the availability of STI services through awareness sessions given in the clinics, usage of Community Health Workers (CHWs), social media platforms, and condom distribution in high-risk areas.

Sub-theme 5.1: Educate during clinic hours

Stakeholders indicated that, early in the clinic’s operations, staff conduct health talks for patients in a group. Additionally, health education talks continued in the consultation rooms, where patients received information face-to-face, individually, with the healthcare providers.

*“We provide individual health education in consultation rooms. We also provide group sessions, covering important topics such as how STIs are transmitted, how they can be treated, and the best ways to prevent them.”*

*(P7, Female, Professional Nurse, 34 years old).*

Sub-theme 5.2: Community Led Programmes (Peer Educators, Home-based Carers)

Stakeholders expressed that each facility has a CHW, including peer counsellors and home-based caregivers who serve the community, making their healthcare services, such as STIs, readily available and accessible.

*“We have peer councillors and home-based carers who conduct campaigns or STI drives mainly targeting the lesbian, gay, bisexual, transgender, questioning, and queer (LGBTQ) community, where condoms and healthcare services are distributed. These services are also available to everyone in the community, including within taverns, schools, and universities.”*

*(P7, Female, Professional Nurse, 34 years old; P11, Female, Clinical Nurse Practitioner, 50).*

Sub-theme 5.3: Patients followed up

Stakeholders involved in STI services reported that patient follow-up was conducted both telephonically and through CHWs, particularly for patients with serious health conditions and those who demonstrated poor adherence to prescribed medication regimens.

*“We follow our clients physically using phones and through CHWs. When CHWs go to the field, they come across other patients who, along the way, did not complete their prescribed medication courses. The CHWs will bring the patient back to the clinic, where they will be given medication again.”*

*(P7, Female, Professional Nurse, 34 years old).*

Sub-theme 5.4: Social media

Stakeholders indicated their use of social media to create awareness of STI services and to ask for assistance from neighbouring facilities.

*“We do outreach activities or awareness programmes using various platforms such as Facebook.”*

*(P12, Female, Facility Manager, 53 years old).*

Theme 6: Success of sexually transmitted infections services in primary healthcare facilities

Most STI stakeholders had reported that the key strengths of the current programme included effective STIs treatment, health education initiatives, and the provision of condoms. Additionally, some noted that STI services are offered alongside other services in the facilities.

Sub-theme 6.1: Integration of health services

The STI stakeholders outlined that they follow HST guidelines, which are the integration of HIV/AIDS, STIs and TB. They emphasise that STIs in the facilities are paired with other programmes and are never treated as independent services.

*“When the patient comes to the clinic having a headache, for example, we screen for all the diseases. We do not treat STI as an isolated disease.” “We have integrated STI services with chronic, acute, family planning, HIV and pregnancy, TB, sexual reproductive health, ANC, Mother and Child Health, and Prevention of Mother-to-Child Transmission.” (PMCT).*

*(P1, Female, Professional Nurse, 60 years old; P18: Female, Director of STI and key populations, 55 years old).*

Sub-theme 6.2: Health education, successful treatment, and condom provision

Stakeholders perceived health education, effective treatment, and sufficient condom distribution as strong points of the STI programme.

*“We have a successful rate of treatment, which is 95% available, health education, and the provision of condoms, necessary guidelines to facilitate this service, and we are available 24 h.”*

*(P5, Female, Facility Manager, 51 years old).*

Theme 7: Facilities challenges

Service providers have identified various daily challenges they encounter in PHC facilities that hinder the provision of quality healthcare services.

Sub-theme 7.1: Staff shortage

The service provider expressed concern about staff shortages in all the PHC facilities. These facilities often see a high volume of patients each day due to their location, e.g., being next to the university, church, and urban areas. As a result, the amount of time staff can dedicate to each patient is limited, potentially leading to a decrease in the quality of the service provided.

*“I do not know how to explain the staffing issue because, according to Workload Indicators of Staffing Needs (WISN), we are enough in this facility, and they allocated staff according to our patients’ headcounts. Our data still says we are okay and do not need more staff members. In reality, according to my view, I will say there is a shortage of staff despite the use of the WISN technique.”*

*(P12, Female, Facility Manager, 53 years old).*

Sub-theme 7.2: Advanced equipment

Service providers have indicated that they primarily rely on HIV/syphilis rapid tests, which only address those two STIs. For other STIs, they use a syndromic management approach, based on guidelines provided by the Department of Health. The issue of funds and the shortage of reagents in the laboratories was also highlighted as a barrier.

*“Yes, sometimes we are told there is no budget for advanced machines. We always require more staff, more equipment, and more reagents. Sometimes, we run out of reagents.”*

*(P17, Female, Lab Manager, 51 years old).*

*“We do not have advanced equipment for testing STIs within PHC facilities; we have a syphilis rapid test. For other STIs, we use the syndromic management approach.”*

*(P1, Female, Professional Nurse, 60 years old).*

Sub-theme 7.3: Patient administration

In the theme below, service providers indicated the challenges they face in the facilities with regard to patient administration.

*“We have challenges with missing files in this facility. You might find that one person ends up having two to three different files, and in this way, we cannot track the client’s medical problem or history.”*

*(P4, Female, Professional Nurse, 39 years old).*

Theme 8: Patient challenges

In this theme, service providers reported challenges they faced in delivering STI treatment, and these included patients’ non-compliance, patients ‘self-diagnosis, wrong addresses, and challenges with partner referrals.

Sub-theme 8.1: Non-compliance of certain clients

Healthcare providers reported that too often they encounter patients not following treatment guidelines

*“Some of our patients are ignorant, while others cannot adapt and correct their lifestyles. They do not use condoms, have multiple partners, and they re-contact STIs and get re-infected.”*

*(P14, Female, Professional Nurse, 30 years old).*

*“The STI-positive clients do not refer their husbands or sexual partners for treatment to the clinic.”*

*(P15, Female, Facility Manager, 31 years old).*

Sub-theme 8.2: Self-diagnosis by the patient

Service providers reported that some of the patients self-diagnose and complicate the treatment course.

*“Our patients primarily rely on social media and Google to self-diagnose. When we provide treatment to such patients, they combine it with medication from the pharmacies, which often leads to a high rate of treatment failure.”*

*(P4, Female, Facility Manager, 51 years old).*

Sub-theme 8.3: Wrong addresses and numbers

Service providers indicated that wrong numbers and addresses prevented proper follow-ups with the patients.

*“Patients tend to give us wrong addresses and numbers; therefore, this makes it difficult to trace them.”*

*(P13, Female, Professional Nurse, 64 years old).*

## 4. Discussion

We assessed the stakeholders’ roles and views regarding the STI programme in terms of its scope, content, quality, availability, affordability, and accessibility of STI services among KPP in PHC facilities in Limpopo Province, South Africa. Our focus also included identifying the gaps and successes in providing these services. Drawing on the pre-assessment WHO checklist tool, STI stakeholders indicated the need for the capacity to analyse the results, political support, and to assess the STI surveillance. The scope of the STI services or programme in PHC facilities was screening, diagnosis, treatment and prevention. To enhance the availability of STI services, the selected PHC facilities in Limpopo Province employed various methods, such as utilising social media, distributing condoms, providing health educational talks during clinic visits, and engaging community-led programmes. To promote accessibility to healthcare services, two facilities operated from Monday to Sunday for 24 h a day and were adequately resourced. Service providers from this study expressed that all the services offered within government PHC facilities were free of charge. To improve the quality of services, service providers relied on in-service training and monthly data monitoring. However, several challenges hindered STI service provision, and these included staff shortages, a lack of advanced equipment, issues with patient administration, and patients’ self-diagnosis. On the other hand, factors that facilitated the delivery of STI services included service integration, a sufficient supply of STI medication and condoms, as well as effective treatment protocols.

### Pre-Assessment Using the WHO STI Checklist Tool

The STI service providers expressed concerns regarding the need for regular STI programme evaluation in PHC facilities and the implementation of assessment outcomes. While evaluations of other programmes are conducted within the facilities, the outcomes of these assessments are seldom acted upon. This lack of action may suggest insufficient support from higher-level management or even political support. Confirming the findings of this study, a Kenyan study also reported similar results among healthcare workers [14]. Meanwhile, the facilities added that the analysis was being conducted by the Department of Health District Office rather than by them. These findings were supported by Garrib and colleagues [28], who discovered that there was minimal data utilisation, analysis, and interpretation at the facility level.

The selected PHC facilities in the current study relied on the use of syndromic management, which is consistent with previous literature [14,29]. In this study, the use of social media was linked to enhanced STI awareness in the selected PHC facilities. This was in line with a scoping review examining the use of social media for sexual health promotion, which reported increased awareness of STI prevention methods [30]. A randomised controlled study revealed that social media encouraged young MSM to utilise HIV test kits in the intervention group, as opposed to a control group [31]. In facilitating and promoting the accessibility of STI services, it was discovered that peer counsellors and home-based carers in the selected facilities are actively distributing condoms and encouraging HIV and pregnancy testing in the communities. Aligned with the latter results, it was previously reported that the CHWs play a vital role in expanding healthcare services, such as chronic disease, antenatal and postnatal care services within PHC facilities [15,32]. Two PHC facilities in this study demonstrated that operating continuously from Monday to Sunday and on a 24-h basis also significantly enhanced access to STI services. A study conducted by the City of Johannesburg demonstrated that extending clinics’ operating hours reduces hospital overcrowding and improved the accessibility of healthcare services for diverse population groups, supporting the findings of this study [33]. In contrast to a study conducted in the eThekwini district of Gauteng Province among young women, a clash between clinic operating hours and school and university schedules was reported. [15]. The author stated that by the time students or learners are back, the clinics would have been closed, thereby limiting access to STI services [15]. The findings of the study may vary due to differences in the study populations and their locations. Furthermore, the PHC facilities improved healthcare accessibility by employing the South African Triage Scale (SASA) method, commonly known as “triage”. The method was implemented as a strategy to address overcrowding by enabling healthcare personnel to prioritise patients according to the severity of their conditions. Supporting these findings, Dixon et al. [34] noted that triage systems in crowded environments, such as prehospital and emergency care, facilitated effective patient sorting based on clinical urgency.

Service providers also engage in peer-led in-service training to improve the quality of the STI services rendered. Voegeli et al. [35] emphasised that longer training programmes are necessary to achieve more consistent and effective outcomes. In-service training for healthcare providers plays a critical role in enhancing service quality and ensuring the accurate dissemination of health information, and equipping healthcare workers with new skills or reinforcing existing competencies [36,37]. Data monitoring was also performed at the facilities to ensure data reporting completeness. This was performed to enhance the quality of data reporting. Similar findings were reported by Oyebola et al. [38], wherein the facility managers audited and monitored the captured data on a monthly basis.

Stakeholders in this study reported several systemic challenges, including staff shortages, overcrowding, and long queues. These findings align with previous research indicating that clinics are understaffed and lack the presence of medical doctors [39]. According to Retshidze [40], these factors may contribute to substandard service delivery and increased risk of misdiagnosis. Similarly, the Ritshidze study reported that, due to staff shortage, patients who arrive at PHC facilities early in the morning are often only attended to late in the afternoon [39]. These delays underscore the urgent need to increase the number of healthcare workers in PHC facilities to ensure the delivery of high-quality healthcare services.

This study also identified challenges related to filing systems in selected PHC facilities, including the misplacement or loss of patients’ files. This is consistent with findings from the Ritshidze study [39], which reported that 40% of public healthcare long waiting times and difficulties in retrieving medical files often stemmed from disorganised or inadequate storage systems. Contributing factors may include insufficient storage space and poor file management practices, which can also result in loss of patients’ files [40].

In the current study, STI stakeholders reported a lack of advanced laboratory equipment necessary for accurate STI diagnosis. As a result, collected samples are often referred to nearby hospitals and external laboratories for further analysis. These findings are consistent with evidence from our systematic review, which revealed that many PHC facilities continue to rely on syndromic management approaches due to insufficient financial resources to support laboratory-based diagnostic techniques [29]. Furthermore, the STI stakeholders working within the laboratories reported using in-house assays, multiplexed PCR, RNA and DNA-based equipment, molecular assays, and serological tests. Despite the availability of these advanced methods, their widespread implementation was hindered by high maintenance costs and inadequate funding, limiting the overall effectiveness and accessibility of STI diagnostic services [29].

This study revealed non-compliance with STI treatment guidelines among some patients, particularly regarding the completion of prescribed treatment courses. Consistent results were described in a study by Tisler-Sala et al. [41], which found that patients treated for chlamydia and gonorrhoea in Estonia faced challenges with treatment compliance. The same study identified multiple influencing factors, which included the patient’s gender, the medical speciality of the prescribing physician, the area of residence, and the patient’s age.

An Iranian study showed that partner notification (PN) or referral was associated with barriers such as stigma, discrimination, and fear of disclosing one’s status to sexual networks [42]. In line with this study, PHC facilities experienced challenges with patient referrals primarily due to the provision of incorrect contact numbers and addresses. These findings are consistent with a study conducted in rural South African clinics among patients receiving antiretroviral therapy [43]. The study reported that clinics used telephone calls to follow up with patients. However, for some individuals, the phone number provided was only effective during the initial contact; subsequent attempts were unsuccessful, as patients often did not respond once they recognised that the calls were from the clinic [43].

Service providers described that the strength of the current STI programme lies in the effectiveness of the STI treatments and their availability, health education, rapid diagnostic testing for HIV/syphilis, MUS, syphilis surveillance, and condom distribution. Moreover, the STI stakeholders in this study reported that service integration is already being implemented, with STI services incorporated alongside family planning, HIV and TB care, sexual and reproductive health, and PMCT programmes. This integrated approach improves the availability and accessibility of STI services and may encourage early screening for STIs. This, in turn, can lead to early diagnosis, prompt treatment, and effective prevention [2]. Additionally, STI service providers in this study reported consistent availability of STI medications and condom supply. This was in contradiction with our systematic review and a study conducted in Kenya, where healthcare workers indicated frequent shortage of STI medication within PHC facilities [14,29]. Various study designs, including systematic reviews and qualitative studies, as well as differences in locations (SSA, Kenya, and South Africa), interview guiding tools, and the consideration of five facilities in the current study, may explain the observed differences. All services offered at government PHC facilities were also reported to be free of charge, further supporting equitable access to STI prevention and treatment services among KPP.

## 5. Limitations and Strengths of the Study

Only five PHC facilities in the Capricorn district, Limpopo Province, were selected to conduct the study. As a result, the findings may not be applicable to other facilities in the district or to facilities in other provinces of the country. The participants in this study were STI stakeholders; future research could benefit from including STI service users to gather their views on current service delivery. While this study focused on PHC facilities, additional research might be valuable if conducted in PHC facilities and hospitals to gain a broader understanding of STI services provision. This research has identified several key findings that are essential for enhancing and improving the STI programme at PHC facilities for KPP. It provides the evidence-based insight necessary for strengthening the response to STI services.

Furthermore, rigorous processes were implemented to validate and verify the selection of the relevant themes, and only knowledgeable and experienced STI stakeholders, each with a minimum of six years of service, participated in the study.

## 6. Conclusions

Findings from the selected PHC facilities indicated that the syndromic management approach was the most effective method currently used to address STIs in the Capricorn District of Limpopo Province. We found that service integration already exists in the selected PHC facilities and can be leveraged to improve health outcomes and access to various healthcare services.

The result of this study confirms that social media is an important platform for raising awareness about STI services. There were several challenges that impacted service delivery in the selected facilities, including staff shortage, inefficient filing systems, a lack of advanced diagnostic resources, and patient noncompliance. To improve the situation, it is essential to increase the number of staff members in the selected PHC facility. This will help reduce overcrowding and long waiting times, thereby ensuring high-quality service.

To strengthen the health system effectively within the selected facilities, we recommend gathering feedback from STI service users regarding their experiences with the current STI service provision in PHC facilities in the Capricorn District of Limpopo Province. This will help better understand their challenges and identify successes in service provision to foster targeted interventions.

## Figures and Tables

**Table 1:** Characteristics of the stakeholders involved in STI service delivery.

STI Stakeholders	Age, (Years)	Gender	Qualifications	Job Title	Years of Service
1	60	Female	Diploma	Professional nurse	24
2	35	Female	Hons degree	Facility manager	10
3	58	Male	Degree	Facility manager	26
4	39	Female	Diploma	Professional nurse	18
5	51	Female	Degree	Facility manager	26
6	46	Female	Gr 12	Professional nurse	22
7	34	Female	Diploma	Professional nurse	7
8	48	Female	Diploma	Professional nurse	31
9	50	Female	Degree	Facility manager	21
10	33	Female	Degree	Professional nurse	7
11	50	Female	Diploma	Professional nurse	25
12	53	Female	Degree	Facility manager	26
13	64	Female	Diploma	Professional nurse	13
14	30	Female	Diploma	Professional nurse	7
15	31	Female	Degree	Facility manager	6
16	47	Female	PhD	Epidemiologist	9
17	51	Female	Diploma	Lab manager	10
18	55	Female	MPH. Degree	Dep director	8

Prof—professional nurse, MPH—Master of Public Health, Dep—deputy, Lab—laboratory manager, Hons—honours, Gr—grade, STI—sexually transmitted infections, PhD—Doctor of Philosophy.

## Data Availability

The transcripts of the study are available upon request from Seloka MA (mohlago9592@gmail.com).

## Abbreviations

The following abbreviations are used in this manuscript:

ANC	Antenatal care
AIDS	Acquired immunodeficiency syndrome
ART	Antiretroviral treatment
CHWs	Community Health Workers
DNA	Deoxyribonucleic acid
GD	Genital discharge
HCW	Healthcare workers
HPV	Human papillomavirus
HIV	Human immunodeficiency virus
KPP	Key and priority populations
LGBTQ	Lesbian, gay, bisexual, transgender, questioning
LP	Limpopo Province
MUS	Male urethritis syndrome
MSM	Men who have sex with men
MPH	Master of Public Health
NICD	National Institute for Communicable Diseases
NG	Neisseria gonorrhoea
NHLS	National Health Laboratory Services
NSP	National Strategic Plan
OPD	Outpatient department
PACER	Pan African Centre for Epidemic
PCR	Polymerase chain reaction
PHC	Primary healthcare
PhD	Doctor of Philosophy
PMCT	Mother and Child Health and Prevention of Mother-to-Child Transmission
PN	Partner notification
REC	Research Ethics Committee
RNA	Ribonucleic Acid
SAMRC	South African Medical Research Council
SASA	South African Triage Scale
STIs	Sexually transmitted infections
TB	Tuberculosis
VMMC	Voluntary Medical Male Circumcision
VDS	Vaginal discharge syndrome
UJ	University of Johannesburg
WHO	World Health Organisation
WISN	Workload Indicators of Staffing Needs
